# A Systematic View Exploring the Role of Chloroplasts in Plant Abiotic Stress Responses

**DOI:** 10.1155/2019/6534745

**Published:** 2019-07-18

**Authors:** Yo-Han Yoo, Woo-Jong Hong, Ki-Hong Jung

**Affiliations:** Graduate School of Biotechnology & Crop Biotech Institute, Kyung Hee University, Yongin 17104, Republic of Korea

## Abstract

Chloroplasts are intracellular semiautonomous organelles central to photosynthesis and are essential for plant growth and yield. The significance of the function of chloroplast-related genes in response to climate change has not been well studied in crops. In the present study, the initial focus was on genes that were predicted to be located in the chloroplast genome in rice, a model crop plant, with genes either preferentially expressed in the leaf or ubiquitously expressed in all organs. The characteristics were analyzed by Gene Ontology (GO) enrichment and MapMan functional classification tools. It was then identified that 110 GO terms (45 for leaf expression and 65 for ubiquitous expression) and 1,695 genes mapped to MapMan overviews were strongly associated with chloroplasts. In particular, the MapMan cellular response overview revealed a close association between heat stress response and chloroplast-related genes in rice. Moreover, features of these genes in response to abiotic stress were analyzed using a large-scale publicly available transcript dataset. Consequently, the expression of 215 genes was found to be upregulated in response to high temperature stress. Conversely, genes that responded to other stresses were extremely limited. In other words, chloroplast-related genes were found to affect abiotic stress response mainly through high temperature response, with little effect on response to drought and salinity stress. These results suggest that genes involved in diurnal rhythm in the leaves participate in the reaction to recognize temperature changes in the environment. Furthermore, the predicted protein–protein interaction network analysis associated with high temperature stress is expected to provide a very important basis for the study of molecular mechanisms by which chloroplasts will respond to future climate changes.

## 1. Introduction

Chloroplasts are cellular organelles in which photosynthesis occurs, and they are found in cyanobacteria, algae, and higher plants [1]. Chloroplasts have a double-celled composite membrane and an extensively folded thylakoid membrane. The chloroplast is divided into two compartments, the soluble stroma and lumen, with the latter enclosed by the thylakoid membrane, which carries chlorophylls and other pigments [2]. Chloroplasts produce the energy needed to sustain life through photosynthesis and oxygen-release processes. The key function of chloroplasts is photosynthesis. However, they also affect the physiology and development of plants by their involvement in the synthesis of amino acids, nucleotides, fatty acids, phytohormones, and vitamins [3]. Furthermore, metabolites synthesized in chloroplasts protect plants from environmental (abiotic) and biotic stresses, including heat, cold, drought, salt, light, and pathogens [4, 5]. Chloroplasts have genomes containing approximately 120 genes, which encode key proteins involved in metabolic processes in green plants [6–8].

**Figure 1:**
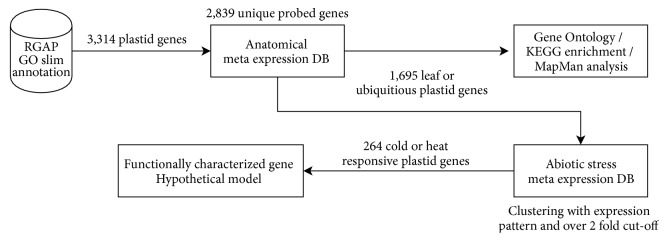
*Workflow diagram summarizing analysis process about rice plastid-related genes*. The workflow illustrates the entire analysis process in this study. First of all, we retrieved 4,707 plastid transcripts from GO slim annotation at the RGAP database. Then we removed unannotated and duplicated information to collect 3,314 plastid genes. By querying these genes to our meta-expression data source, we obtained 2,839 genes that only have the most highly expressed probe. We clustered these intensity values with KMC algorithm as 20 clusters. As a result, we identified 1,695 leaf-preferred or ubiquitously expressed genes for further analysis. With these two sets of genes, we performed functional characterization like GO enrichment, KEGG enrichment, and MapMan analysis to characterize their functions. In addition to these analyses, we queried the 1,695 genes to an abiotic stress expression database (DB) to identify stress-responsive plastid genes. As a result, we clustered 264 cold or heat stress-responsive plastid genes and conducted a literature search. Altogether, we constructed a hypothetical protein–protein interaction model of stress-related plastid genes.

The role of chloroplasts in plant abiotic stress responses continues to be highlighted. The development of high-throughput sequencing technologies has made possible many advances in plant genetics and genomics. In this study, we tried a new approach through transcriptome analysis of chloroplast-related genes. Initially, we selected 3,314 plastid-related genes via Rice Genome Annotation Project (RGAP) Gene Ontology (GO) slim annotation. Then, we used the anatomical meta-expression database and, as a result, identified 1,695 leaf-preferred or ubiquitously expressed plastid-related genes. Subsequently, GO enrichment, Kyoto Encyclopedia of Genes and Genomes (KEGG) enrichment, and MapMan analyses of these 1,695 genes were performed and significant functional groups were identified. Of the 1,695 leaf-preferred or ubiquitously expressed plastid-related genes, meta-expression analysis under abiotic stress conditions identified 264 cold or heat stress-responsive plastid-related genes. The analysis of functionally characterized plastid-related genes emphasized the significance of our candidate genes for crosstalk between chloroplast development and heat stress. Ultimately, the hypothetical network model will provide a valuable backbone for future studies (Figure 1).

## 2. Materials and Methods

### 2.1. Integration of Plastid Genes from Public Data Source

We obtained information on plastid-related genes from the GO slim annotation data at the Rice Genome Annotation Project web database (RGAP) [9]. We first selected 3,314 plastid-related genes in the cellular component subontology from the data. Then, we removed duplicated loci because of their transcript isoforms. Finally, we collected 3,314 plastid-related genes for further analysis.

### 2.2. Collection of Microarray Data

We collected transcriptomics data to analyze anatomical and abiotic expression patterns of 1,696 plastid-related genes, for which we used the data source described in our previous report [10]. Detailed information is as below. For analysis of anatomical expression profiles, we integrated anatomical data from the rice oligonucleotide array database ROAD [11]. For the abiotic stress database, we retrieved 13 expression dataset series, GSE16108, GSE18930, GSE21651, GSE23211, GSE24048, GSE25176, GSE26280, GSE28209, GSE31077, GSE33204, GSE37940, GSE38023, and GSE6901 from the National Center for Biotechnology Information Gene Expression Omnibus (https://www.ncbi.nlm.nih.gov/gds), and one data series, E-MEXP-2401, from the European Bioinformatics Institute (EMBL-EBI) ArrayExpress database (https://www.ebi.ac.uk/arrayexpress/).

### 2.3. Clustering of Transcriptome Data

MeV (*M*ultiple* E*xperiment* V*iewer) is a widely used program to visualize transcriptome data and to perform statistical analysis [12]. We used MeV software (version 4.9.0) to visualize our microarray data. For analysis of transcriptome data, we applied a k-means clustering (KMC) algorithm with Euclidean distance metric embedded in MeV with the same method as that used for the identification of late-pollen-preferred genes in rice [13]. In the case of the abiotic microarray data, we clustered 1,695 leaf-preferred or ubiquitously expressed plastid genes with the same KMC algorithm and matrix. In addition, we selected only genes with an average log_2_-fold-change value (treatment/control) in a cluster greater than 1(log_2_⁡value) and a p-value of one-way analysis of variance (ANOVA) of less than 0.05 to ensure clear correlation. We used the row.oneway.anova function in the HybridMTest package in R to perform a one-way ANOVA test and used Illustrator software (Adobe Illustrator CS6) to present heatmap images [14].

### 2.4. GO Enrichment Analysis

GO enrichment is commonly used to interpret functional roles of large-scale transcriptomics data [15]. In the current study, we used the ROAD database to find GO terminology for each cluster (http://ricephylogenomics-khu.org/ROAD_old/analysis/go_enrichment.shtml, temporary homepage for updating). To perform GO enrichment analysis, we applied the following criteria: query number of >2, hyper p-value of <0.05, and fold-enrichment value (query number/query expected number) of >2, by referencing previous reports about GO enrichment analysis [13, 16]. We selected significant GO terms and integrated cluster information from transcriptomic data analysis with each selected GO term. Finally, we visualized these data via R Studio (version 1.1.453) and ggplot2 R package (version 3.0.0) [17].

### 2.5. KEGG Enrichment Analysis

We performed KEGG enrichment analysis using R Studio and the clusterProfiler package [18]. To use the enrichKEGG function in this package, we used input data consisting of cluster information and rice annotation project database ID. In addition, we chose data for organism code and filtered out results by applying adjusted p-value cut-offs of less than 0.05, as in other studies [19, 20]. For visualization of the results, we used dotplot function in the package and modified the figure with the ggplot2 package (version 3.0.0).

### 2.6. MapMan Analysis

To obtain a systemic view of the 1,695 leaf-preferred and ubiquitously expressed plastid-related genes within rice, we performed MapMan analysis as previously described [21]. In detail, we conducted the functional characterization of the genes by uploading the locus list with cluster information to MapMan software (v3.6.0 RC1) [22]. Among the various functional classifications, we analyzed the metabolism, cellular response, regulation, and transcription factor overviews in detail (Figure 3).

### 2.7. Analysis of Functionally Characterized Genes via Literature Search

To identify the previously characterized functional roles of the 264 plastid-related genes associated with heat or cold stresses, we used the funRiceGenes database for functionally characterizing rice genes (https://funricegenes.github.io/) [23]. In this database, information on 3,148 functionally characterized genes is available. As in our previous study [24], we parsed functional roles for the 264 genes and gene clusters, using meta-expression data. Subsequently, we summarized the data in Table 1, using Excel software.

### 2.8. Analysis of a Predicted Protein–Protein Interaction Network

Using the Rice Interactions Viewer tool (http://bar.utoronto.ca/interactions/cgi-bin/rice_interactions_viewer.cgi), we generated a hypothetical protein–protein interaction network, involving transcription factors (TFs), redox reactions, and functionally characterized genes. The network was edited with the Cytoscape tool (3.2.0 version) [25].

## 3. Results

### 3.1. Integration of 3,314 Plastid-Related Genes from GO Slim Annotation at the Rice Reference Database, RGAP

We applied GO slim annotation at a rice reference database, RGAP. GO provides controlled information on each gene, which classifies the genes into three categories, namely, biological process, molecular function, and cellular component [26]. We retrieved 4,707 plastid-related transcripts with the cellular component information of GO. Then, we selected only chromosome-annotated genes without duplication. As a result, we selected 3,314 genes for further analysis.

### 3.2. Anatomical Dissection of Chloroplast-Related Genes via Meta-Expression Analysis

**Figure 2:**
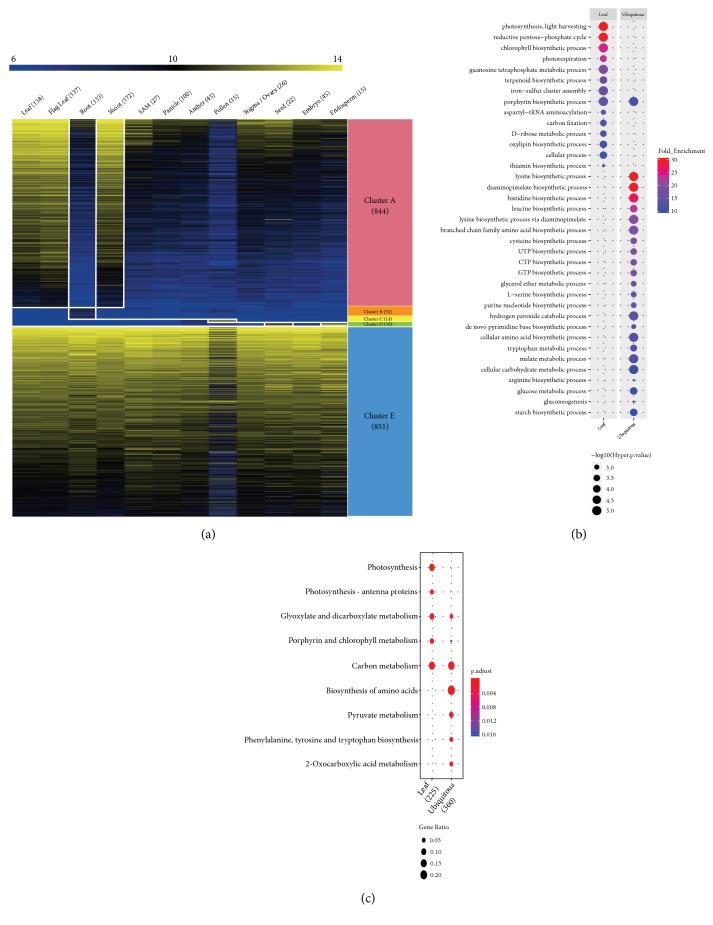
*Meta-expression profile and functional analysis of the 1,695 leaf-preferred or ubiquitously expressed genes*. We performed meta-expression analysis with large-scale microarray dataset for elucidating tissue-specific patterned plastid genes. In addition, we performed GO and KEGG enrichment analysis to identify functional roles for two clusters, leaf-preferred cluster A and ubiquitous cluster E. (a) Heatmap analysis of plastid-related genes and identification of five clusters. We performed KMC clustering into 20 clusters using Euclidean distance matric and selected 10 clusters on the basis of tissue-specific expression patterns. Among these clusters, we selected two major clusters, A (leaf-preferred genes) and E (ubiquitous genes) for further functional enrichment analysis. Digits under or beside each clusters indicate number of the genes that were classified into each cluster. (b) GO enrichment analysis of 1,695 leaf-preferred and ubiquitous expressed genes. To reveal characteristics of each cluster, we conducted GO enrichment analysis and visualized the result with ggplot2 package. GO terms were classified according to biological process GO terms. Dot color indicates fold-enrichment value (blue color is 2-fold, which is the minimum cut-off to select significant fold-enrichment value, and red color indicates higher fold-enrichment value greater than two), and dot size indicates statistical significance (-log_10_⁡(hyper  p-values) are used, with higher values having greater significance). (c) KEGG enrichment analysis of two clusters, A and E. Enriched KEGG pathway indicated with dot size representing the ratio of selected genes to total genes in the pathway and dot color illustrating adjusted p-value. The numbers below clusters indicate the number of mapped genes to selected KEGG pathways.

To assess the functional roles of 3,314 plastid-related genes, we used meta-anatomical expression profiles consisting of 983 rice Affymetrix array anatomical sample data [10]. By applying clustering analysis based on the Euclidian distance algorithm, we grouped 2,839 genes with probes on the Affymetrix array into 20 anatomical clusters (Figure S1). Based on this analysis, we identified plastid-related genes, with respect to organ-selective expression in leaves or ubiquitous expression pattern. For example, cluster A, with 844 genes, had preferential expression in leaves and cluster E, with 851 genes, exhibited an ubiquitous expression pattern, which was related to housekeeping functions (Figure 2(a)). In addition, we found that clusters B and C were closely associated with roots (52 genes) and pollen (14 genes), respectively, and cluster D with seed (16 genes).

### 3.3. Functional Enrichment Analysis of Plastid-Related Genes with Leaf-Preferred or Ubiquitous Expression Using Gene Ontology and KEGG

We focused on plastid-related genes in two clusters associated with the leaf or all organs (ubiquitously expressed genes) through meta-expression analysis. We then performed a functional-group enrichment analysis for each of the two anatomical clusters. To determine significant functional groups associated with the two anatomical clusters, we performed GO and KEGG enrichment analyses (Figures 2(b) and 2(c)). In all, 45 GO terms in the biological process category were highly overrepresented in the leaf gene list (cluster A), with p-values of <0.05 and (log_2_)-fold-enrichment values of >2, as we previously reported [27]. They included biological processes related to photosynthesis, namely, light harvesting (36.3-fold enrichment, GO:0009765), reductive pentose-phosphate cycle (30.9, GO:0019253), chlorophyll biosynthetic process (23.9, GO:0015995), photorespiration (23.2, GO:0009853), guanosine tetraphosphate metabolic process (19.3, GO:0015969), terpenoid biosynthetic process (15.4, GO:0016114), iron-sulfur cluster assembly (15.4, GO:0016226), aspartyl-tRNA aminoacylation (14.5, GO:0006422), porphyrin biosynthetic process (14.5, GO:0006779), D-ribose metabolic process (11.9, GO:0006014), carbon fixation (11.9, GO:0015977), oxylipin biosynthetic process (11.4, GO:0031408), cellular process (10.7, GO:0009987), and thiamin biosynthetic process (10.5, GO:0009228) (Figure 2(b)). The 31 GO terms with fold-enrichment values less than 10 are shown in Figure S2. Similar to the results from GO enrichment analysis, KEGG enrichment also showed that photosynthesis, photosynthesis−antenna proteins, glyoxylate and dicarboxylate metabolism, porphyrin and chlorophyll metabolism, and carbon metabolism were enriched in leaves (Figure 2(c)). These results suggest that plastid-related genes with high expression levels in leaves are closely related to photosynthesis, as would be expected.

Next, we performed GO enrichment and KEGG enrichment analyses on ubiquitously expressed genes. As a result, we found that 65 GO terms were enriched in cluster E: lysine biosynthetic process (30.8-fold enrichment, GO:0009085), diaminopimelate biosynthetic process (30.8, GO:0019877), histidine biosynthetic process (27.4, GO:0000105), leucine biosynthetic process (23.0, GO:0009098), lysine biosynthetic process via diaminopimelate (20.5, GO:0009089), branched chain family amino acid biosynthetic process (19.6, GO:0009082), GTP biosynthetic process (18.5, GO:0006183), UTP biosynthetic process (18.5, GO:0006228), CTP biosynthetic process (18.5, GO:0006241), cysteine biosynthetic process (18.5, GO:0019344), purine nucleotide biosynthetic process (15.3, GO:0006164), L-serine biosynthetic process (15.3, GO:0006564), glycerol ether metabolic process (15.3, GO:0006662), hydrogen peroxide catabolic process (14.2, GO:0042744),* de novo* pyrimidine base biosynthetic process (13.2, GO:0006207), cellular amino acid biosynthetic process (12.5, GO:0008652), tryptophan metabolic process (12.3, GO:0006568), malate metabolic process (12.0, GO:0006108), porphyrin biosynthetic process (11.5, GO:0006779), glucose metabolic process (10.3, GO:0006006), gluconeogenesis (10.3, GO:0006094), arginine biosynthetic process (10.3, GO:0006526), starch biosynthetic process (10.3, GO:0019252), and cellular carbohydrate metabolic process (10.3, GO:0044262) (Figure 2(b)). The 41 GO terms with fold-enrichment values less than 10 are shown in Figure S2. Consistent with the GO enrichment analysis results, KEGG enrichment also showed that amino acid biosynthesis, including that of phenylalanine, tyrosine, and tryptophan, carbon metabolism, pyruvate metabolism, and 2-oxocarboxylic acid metabolism were enriched in all plants (Figure 2(c)). These results suggest that plastid-related genes with ubiquitous expression are closely related to basic metabolic processes.

### 3.4. Analysis of Various MapMan Overviews Associated with Plastid-Related Genes with Leaf-Preferred or Ubiquitous Expression

**Figure 3:**
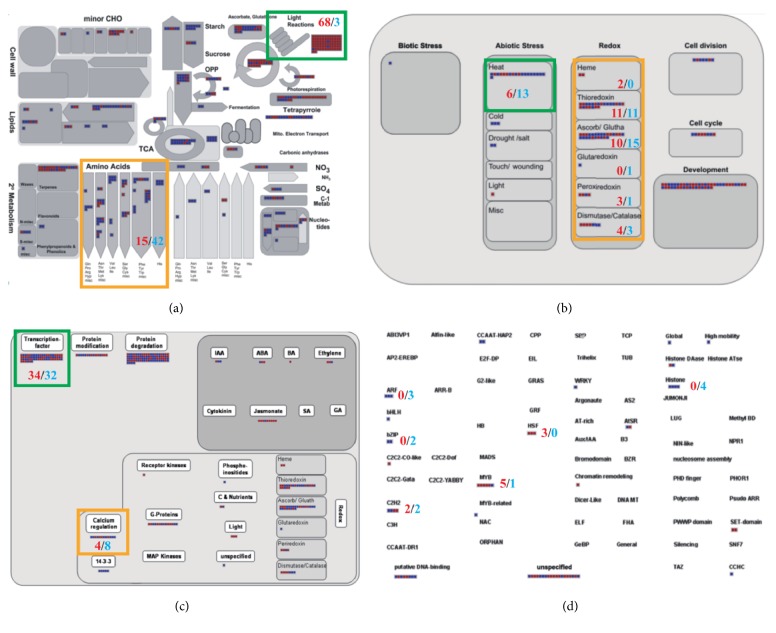
*Functional classification of the leaf-preferred or ubiquitously expressed plastid genes via MapMan analysis*. MapMan analysis of the two clusters for functional classification within various biological processes. (a) Metabolism overview, (b) cellular response overview, (c) regulation overview, and (d) transcription factor overview. Red and blue squares indicate members of leaf-preferred and ubiquitously expressed clusters, respectively. In addition to squares, red and blue digits show number of squares. Green box and orange box in metabolism, cellular response, and regulation overview highlight areas that are discussed in the Results.

The MapMan program is an effective tool for visualizing diverse overviews associated with high-throughput transcriptome data [28]. We uploaded plastid-related locus IDs for the 844 upregulated genes in leaves (Figure 3, red squares) and the 851 ubiquitously expressed genes (Figure 3, blue squares). In the metabolism overview, we observed that genes with increased expression in leaves were mainly found to be involved in light reactions (71 elements, 68 for leaf-preferred expression/3 for ubiquitous expression, i.e., 68/3), whereas those with ubiquitous expression were found to be largely involved in amino acid metabolism (57 elements, 15/42) (Figure 3(a), green and orange box). Most of the light reactions were found to be associated with leaf-preferred genes, whereas amino acid metabolism was mainly identified in ubiquitously expressed genes. Interestingly, these results are consistent with the findings of the GO enrichment and KEGG enrichment analyses.

In the cellular response overview, heat stress within abiotic stress response was closely associated with both leaf-preferred or ubiquitously expressed genes (19 elements, 6/13) (Figure 3(b), green box). Many antioxidant proteins such as thioredoxin, ascorbate, glutathione, and peroxiredoxin and catalases were found in the redox cellular response (61 elements, 30/31) (Figure 3(b), orange box), with leaf-preferred or ubiquitously expressed genes being identified in similar numbers in heat stress and redox responses. Independent of organ specificity, these results suggest that plastid-related genes are closely related to heat stress and utilize proteins catalyzing redox reactions to effectively remove superoxide and H_2_O_2_ produced under stressful conditions.

Finally, we identified leaf-preferred or ubiquitously expressed genes associated with TFs (66 elements, 34/32) and calcium regulation (12 elements, 4/8) in the regulation overview (Figure 3(c), green and orange box). As a result of detailed examination of TFs, we found six (5/1) myeloblastosis (MYB) oncogenes, four (0/4) histones, four (2/2) Cys2His2 (C2H2) zinc fingers, three (0/3) auxin-response factors (ARFs), three (3/0) heat shock TFs (HSFs), and two (0/2) basic leucine zipper (bZIP) TFs (Figure 3(d)). Most of the MYB and HSFs were found in leaf-preferred genes, and histone, ARFs, and bZIP TFs were mainly identified in ubiquitously expressed genes. We have identified various TFs and calcium regulation elements in Figures 3(c) and 3(d). These results suggest that Ca^2+^ is involved in the activation of genes encoding HSFs and heat shock proteins (HSPs) during heat stress.

### 3.5. Abiotic Stress Dissection of Plastid-Related Genes via Meta-Expression Analysis

**Figure 4:**
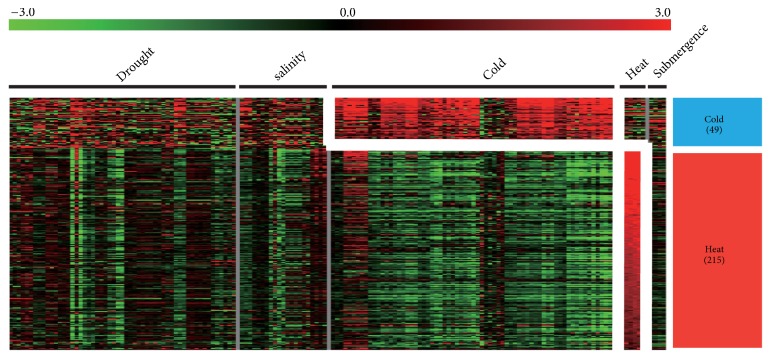
*Heatmap analysis of cold- and heat-responsive plastid genes which show leaf-preferred or ubiquitous expression patterns*. Heatmap of cold or heat stress-responsive plastid-related genes. Similarly to the anatomy clustering, we used KMC algorithm with Euclidean distance matric to cluster abiotic-responsive genes. To define stress responsiveness, we applied criteria that were greater than average 1 log_2_-fold change (2-fold) in each stress and p-value less than 0.05 in one-way ANOVA test. As a result, we identified 264 cold or heat stress-responsive plastid genes.

In the previous section, we used MapMan analysis to deduce that plastid-related genes were closely associated with heat stress response. To test this hypothesis, we conducted meta-expression analysis under abiotic stress conditions, such as drought, salinity, cold, heat, or submergence, for 1,695 genes (844 genes with preferential expression in leaves and 851 genes with ubiquitous expression), using meta-expression data (Figure S3). As a result, 215 genes were associated with heat stress and 49 genes with cold stress (Figure 4). However, we did not identify candidate genes showing differential expression patterns clearly associated with drought, salinity, or submergence. These results suggest that plastid-related genes respond mainly to changes in external temperature rather than to other stresses.

### 3.6. Evaluation of Candidate Genes Associated with Plastid-Related Genes Using Rice Genes with Known Functions

To evaluate the functional significance of the 215 genes with increased expression under heat stress, we searched the literature to determine what functions of heat stress-responsive plastid genes have been identified in previous studies [23]. Of six genes found in that database (Table 1), five have been linked to various biotic and abiotic stress responses in rice. They include* heat shock protein 90* [29] for environmental stresses;* OsTRXZ* [30] for cold stress;* OsCNX* [31], and* OsCP12* [32] for drought tolerance; and* OsDR8* [33] for disease resistance. Moreover, 12 of the genes are associated with morphological traits:* OsNUS1 *[34],* TCD11 *[35],* TCD5 *[36],* VYL *[37],* WSP1 *[38], and* ZN* [39] with leaf development;* GRY79* [40],* OsCPn60a1* [41], and* OsPDIL1;1* [42] with seedling development;* SPP* [43] with root development;* OsNADH-GOGAT2* [44] with spikelet number; and* OIP30* [45] with pollen trait. In addition, nine genes are associated with physiological traits:* OsClpP5 *[46],* OsPAPST1 *[47],* OspTAC2 *[48],* OsValRS2 *[49], and* OsFLN2 *[50] with chloroplasts;* OsPK2* [51] with grain; OsNADP-ME2 [52] with growth;* RNP29* [53] for phosphate; and* Pho1* [54] for starch.

As expected, most of the heat stress-responsive genes having functions involved in leaf or seedling development were associated with morphological traits, whereas most of the genes associated with physiological traits were related to chloroplasts. Interestingly, in genes associated with abiotic stress responses, more were reported to have functions related to drought, high salinity, and cold stress than with heat stress. For example, the 90 kDa heat shock protein (rHsp90) accumulates after exposure to abiotic stresses such as high salinity, desiccation, and high pH as well as high temperature, and tobacco transgenic plants overexpressing rHsp90 exhibit increased tolerance to salinity [29]. In addition,* temperature-sensitive virescent* (*tsv*) showed defective chloroplasts and decreased chlorophyll content under cold stress. Interestingly, TSV, interacting with OsTRXZ (a subunit of plastid-encoded RNA polymerase (PEP) in chloroplasts), enhanced OsTRXZ stability at low temperatures. These results suggest that plastid-related genes are also associated with various abiotic stresses.

### 3.7. Analyses of Predicted Protein–Protein Interactions Associated with Chloroplast-Related Genes

**Figure 5:**
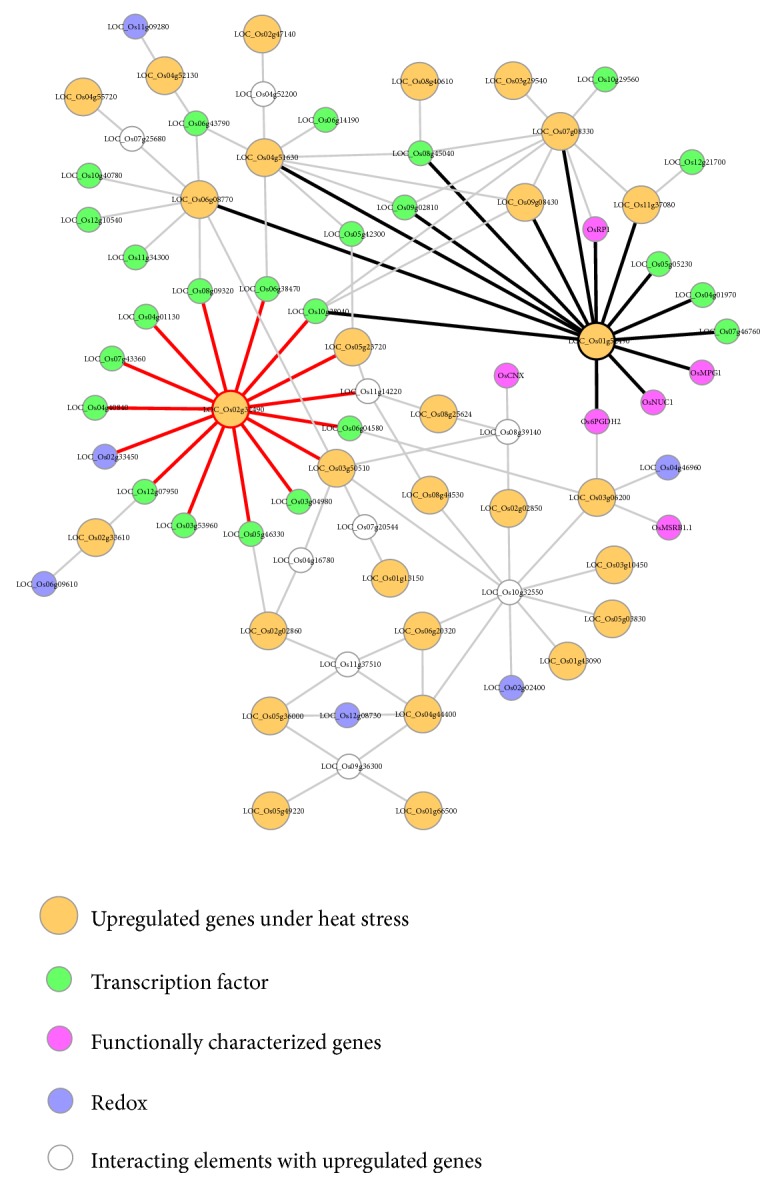
*Construction of regulatory network associated with genes upregulated under high temperature*. Using Rice Interaction Viewer and Cytoscape tools, we queried the predicted protein–protein interaction network associated with 29 upregulated genes under heat stress (orange circles), 24 transcription factors (green circles), six functionally characterized genes (purple circles), and six redox proteins (blue circles).

Regulatory genes are primary targets when investigating diverse stress responses and developmental processes. Understanding the regulatory relationship between them can lead to a new strategy for the manipulation of chloroplasts to improve plant tolerance to heat stress. To expand our knowledge of this mechanism, we utilized the Rice Interactions Viewer to generate a hypothetical protein–protein interaction network associated with the 215 upregulated genes associated with heat stress response [55]. We then refined the network by using genes in the following four categories as the query: 29 plastid-related genes with elevated expression in heat stress (orange circles, Figure 5), 24 TFs (green circles), six functionally characterized genes (purple circles), and six redox proteins (blue circles).

We found two interesting genes in this network.* LOC_Os02g32490*, encoding the AMP-binding enzyme, is predicted to interact with 11 TFs and one involved in the redox reaction (red lines, Figure 5). Another gene,* LOC_Os01g52490*, encoding a 40S ribosomal protein, is likely to be associated with four functionally characterized genes and six TFs (black lines, Figure 5). The four functionally characterized genes are all associated with response to abiotic stress. For example,* OsCTR1*, encoding the RING Ub E3 ligase, interacts with two chloroplast-localized proteins (OsCP12 and OsRP1) and is involved in drought tolerance [32]. The transcript level of* Os6PGDH2*, encoding 6-phosphogluconate dehydrogenase (6PGDH), increases under drought, cold, and high salinity conditions and in response to abscisic acid treatments, under which conditions 6PGDH activity also increases [56]. Finally, the shorter Nucleolin1 gene (*OsNUC1-S*) reduces oxidative stress during high salinity treatment [57].

## 4. Discussion

### 4.1. Chloroplasts Maintain Reactive Oxygen Species (ROS) Homeostasis through Redox Enzymes Such as Superoxide Dismutase (SOD) and the Ascorbate-Glutathione (ASC-GSH) Cycle during Heat Stress

We found that chloroplast-related genes were associated with heat stress under abiotic stress conditions by MapMan analysis (Figure 3(b), green box). In addition, several antioxidants such as thioredoxin, ascorbate, glutathione, peroxiredoxin, and catalases, which are known to play an important role in scavenging ROS, have also been found (Figure 3(b), orange box). ROS are chemically reactive species and products of aerobic metabolism [58]. ROS are mainly produced by chloroplasts, mitochondria, and peroxides and are scavenged by the antioxidant mechanisms [59]. The balance between ROS generation and ROS scavenging is disturbed by various abiotic stresses like extreme temperatures, high salinity, drought, and heavy metals [5]. The plant has developed two efficient antioxidant mechanisms to maintain the ROS homeostasis of cells [5]: (i) antioxidant enzymes, like superoxide dismutase (SOD), ascorbate peroxidase (APX), catalase (CAT), guaiacol peroxidase (GPX), glutathione reductase (GR), dehydroascorbate reductase (DHAR), and monodehydroascorbate reductase (MDHAR); (ii) nonenzymatic antioxidants, such as ascorbic acid (AA), reduced glutathione (GSH), *α*-tocopherol, carotenoids, and flavonoids [58].

The chloroplast utilizes a well-organized thylakoid membrane system to efficiently capture light [60]. In thylakoids, photosystem I (PSI) and photosystem II (PSII) play a key role in the light harvesting system and are the major sources of ROS production. However, abiotic stresses such as drought, high salinity, and temperature extremes induce the formation of O^•−^_2_ in the photosystem through the Mehler reaction. Subsequently, a membrane-bound Cu/Zn SOD at the PSI converts O^•−^_2_ into H_2_O_2_ [58], which is then converted to H_2_O* via* the ascorbate-glutathione (ASC-GSH) cycle [61]. One of the integral components of the ASC-GSH cycle, APX, reduces H_2_O_2_ to H_2_O and DHA, using AA as a reducing agent in the chloroplast [62]. Another key component, GR, is a flavoprotein oxidoreductase, mainly found in chloroplasts. GR uses NADPH to reduce GSSG (glutathione, oxidized form) to GSH, with GSH reacting with and quenching detrimental ROS species such as ^1^O_2_ and OH^•^ [63].

This ROS scavenging by antioxidant enzymes or nonenzymatic antioxidants protects plants from heat-induced oxidative stress. For example,* Zea mays* plants showed greater expression of enzymatic antioxidants, such as CAT, APX, and GR, compared with* O. sativa* and maintained significant levels of nonenzymatic antioxidants such as AA and GSH at high temperatures (45°C for day/40°C for night). These results suggest that* Z. mays* will be better able to cope with oxidative damage by heat stress through the ROS- scavenging process than its fellow grass,* O. sativa* [64]. Furthermore, heat-acclimated turf grass was found to maintain low ROS levels by increasing the synthesis of AA and GSH at high temperatures [65]. These results suggest that the antioxidant defense mechanism plays an important role in heat stress tolerance [66]. We have identified various enzymatic or nonenzymatic antioxidants in chloroplast-related genes through MapMan analysis (Figure 3(b)), and these candidate genes can be used as a major route to develop crops tolerant to abiotic stress, including heat stress.

### 4.2. Transcriptional Activity in the Nucleus Is Partially Regulated by Signals Derived from the Plastids

Chloroplast retrograde signaling refers to a communication pathway in which transcriptional activities in the nucleus are partially regulated by signals derived from plastids [67]. In general, chloroplasts of plants are predicted to be descendants of ancient photosynthetic bacteria and have circular genomes for their transcription and translation machinery [68]. The first discovery of retrograde signaling was reported in barley. Two barley chloroplast ribosome-deficient mutants caused downregulation of nuclear-encoded plastid proteins due to a defect in plastid function [69]. After that, studies on the function of retrograde signaling were conducted by coordinating chlorophyll biosynthesis with the expression of nuclear genes in some plants [70, 71]. Conversely, genome-uncoupled mutants, in which communication between the chloroplast and the nucleus was disrupted, expressed nuclear-encoded photosynthetic genes despite defective chloroplast physiology or inhibited biogenesis [70].

In chloroplast retrograde signaling, heat shock proteins (HSPs) play an important role in heat stress [4]. Interestingly, we identified a number of TFs, including heat shock transcription factors (HSFs), in Figure 3(d). Heat shock genes (HSGs), encoding HSPs, are upregulated during heat stress. HSPs play a role as chaperones to prevent denaturation of intracellular proteins and to preserve stability through protein folding [72]. Under heat stress conditions, plant HSP expression is rapidly activated by specific HSFs binding to the conserved sequences of the heat shock elements in the promoters of heat-responsive genes [73, 74]. For example, H_2_O_2_ in rice increased tolerance to oxidative stress by inducing the expression of chloroplast-localized small HSPs [75]. In addition, high H_2_O_2_ in Arabidopsis has been shown to induce activation of some chaperones, HSPs, and HSFs at the mRNA levels [76]. Until now, the retrograde signals have been perceived only in the cytosol, and the mechanism of communication with the nucleus remains largely unknown. We found several HSFs and other TFs in chloroplast-related genes through MapMan analysis. These might be potential candidate TFs to mediate plastid-nucleus signaling.

## 5. Conclusions

Chloroplasts are sensitive to environmental changes and have developed a complex network of plastid signals to protect plants from environmental stresses. ROS is a by-product of aerobic metabolism and acts as a marker under environmental stress. Chloroplast retrograde regulation is essential for coordinating gene expression, including that involving photosynthesis, in both the nucleus and the chloroplast. To cope with environmental stress, retrograde signals derived from chloroplasts must be delivered rapidly by cytosolic messengers or by distinct signal cascade pathways to the nucleus. H_2_O_2_, the HSP-associated complex, and some TFs have been suggested as possible retrograde signaling molecules, but more research remains to be conducted to determine how they function [4]. We performed functional dissection of chloroplast-related genes via diverse meta-expression data analysis based on microarray data and functional classification analyses. This work might provide new insights into the role of chloroplasts in producing crop plants with enhanced abiotic stress tolerance.

## Figures and Tables

**Table 1:** Summary of functionally characterized plastid genes associated with cold and abiotic stress.

Category^a^	Tissue^b^	Abiotic^c^	Locus_ID	Gene symbol	Keyword^d^	Title^e^
BA^f^	Ubi	Heat	LOC_Os06g50300	Hsp90|rHsp90|OSGrp94	abiotic stress	rHsp90 gene expression in response to several environmental stresses in rice (*Oryza sativa* L.)
BA	Leaf	Heat	LOC_Os08g29110	*OsTRXZ*	cold stress	TSV, a putative plastidic oxidoreductase, protects rice chloroplasts from cold stress during development by interacting with plastidic thioredoxin Z.
BA	Ubi	Heat	LOC_Os04g32950	*OsCNX*	drought tolerance	Heterologous expression of rice calnexin (OsCNX) confers drought tolerance in *Nicotiana tabacum*
BA	Leaf	Heat	LOC_Os03g19380	*OsCP12*	drought tolerance	The rice RING E3 ligase, OsCTR1, inhibits trafficking to the chloroplasts of OsCP12 and OsRP1, and its over-expression confers drought tolerance in *Arabidopsis*
BA	Ubi	Heat	LOC_Os07g34570	*OsDR8*	disease resistance	Dual function of rice OsDR8 gene in disease resistance and thiamine accumulation

MT^g^	Leaf	Heat	LOC_Os03g45400	*OsNUS1|V1*	leaf	A virescent gene V1 determines the expression timing of plastid genes for transcription/translation apparatus during early leaf development in rice
MT	Leaf	Heat	LOC_Os12g37610	*TCD11*	leaf	The rice TCD11 encoding plastid ribosomal protein S6 is essential for chloroplast development at low temperature
MT	Leaf	Heat	LOC_Os05g34040	*TCD5|CSV1|TSV*	leaf	Temperature-sensitive albino gene *TCD5,* encoding a monooxygenase, affects chloroplast development at low temperatures.
MT	Leaf	Heat	LOC_Os03g29810	*VYL*	leaf	A rice virescent-yellow leaf mutant reveals new insights into the role and assembly of plastid caseinolytic protease in higher plants
MT	Leaf	Heat	LOC_Os04g51280	*WSP1*	leaf	The RNA editing factor WSP1 is essential for chloroplast development in rice.
MT	Leaf	Heat	LOC_Os06g02580	*ZN*	leaf	ZEBRA-NECROSIS, a thylakoid-bound protein, is critical for the photoprotection of developing chloroplasts during early leaf development
MT	Leaf	Heat	LOC_Os02g33610	*GRY79*	seedling	*GRY79* encoding a putative metallo-*β*-lactamase-trihelix chimera is involved in chloroplast development at early seedling stage of rice.
MT	Leaf	Heat	LOC_Os12g17910	*OsCPn60alpha1|OsCPn60a1*	seedling	*OsCpn60alpha1*, encoding the plastid chaperonin 60alpha subunit, is essential for folding of rbcL
MT	Ubi	Heat	LOC_Os11g09280	*OsPDIL1;1*	seedling	Formation of protein disulfide bonds catalyzed by *OsPDIL1;1* is mediated by microRNA5144-3p in rice.
MT	Ubi	Heat	LOC_Os06g41990	*SPP*	root	A rice stromal processing peptidase regulates chloroplast and root development
MT	Leaf	Heat	LOC_Os05g48200	*OsNADH-GOGAT2*	spikelet number	Disruption of a novel NADH-glutamate synthase2 gene caused marked reduction in spikelet number of rice
MT	Ubi	Heat	LOC_Os06g08770	*OIP30*	pollen	OIP30, a RuvB-like DNA helicase 2, is a potential substrate for the pollen-predominant OsCPK25/26 in rice

PT^h^	Ubi	Heat	LOC_Os03g19510	*OsClpP5*	chloroplast	An active DNA transposon nDart causing leaf variegation and mutable dwarfism and its related elements in rice
PT	Ubi	Heat	LOC_Os01g16040	*OsPAPST1*	chloroplast	Identification of a dual-targeted protein belonging to the mitochondrial carrier family that is required for early leaf development in rice
PT	Ubi	Heat	LOC_Os03g60910	*OspTAC2*	chloroplast	*OspTAC2* encodes a pentatricopeptide repeat protein and regulates rice chloroplast development.
PT	Ubi	Heat	LOC_Os07g06940	*WP1|OsValRS2*	chloroplast	WHITE PANICLE1, a Val-tRNA synthetase regulating chloroplast ribosome biogenesis in rice, is essential for early chloroplast development.
PT	Leaf	Heat	LOC_Os03g40550	*OsFLN2|HSA1*	chloroplast	FRUCTOKINASE-LIKE PROTEIN 1 interacts with TRXz to regulate chloroplast development in rice.
PT	Ubi	Heat	LOC_Os07g08340	*OsPK2*	grain	*OsPK2* encodes a plastidic pyruvate kinase involved in rice endosperm starch synthesis, compound granule formation and grain filling.
PT	Ubi	Heat	LOC_Os01g52500	*NADP-ME2|OsNADP-ME2*	growth	Expression of an NADP-malic enzyme gene in rice (*Oryza sativa* L.) is induced by environmental stresses; over-expression of the gene in *Arabidopsis* confers salt and osmotic stress tolerance
PT	Leaf	Heat	LOC_Os07g43810	*RNP29*	phosphate	Identification and characterization of chloroplast casein kinase II from *Oryzasativa* (rice).
PT	Ubi	Heat	LOC_Os03g55090	*Pho1*	starch	Mutation of the plastidial alpha-glucan phosphorylase gene in rice affects the synthesis and structure of starch in the endosperm

^a^ Agronomic traits associated with functionally characterized genes out of candidate genes in this study.

^b^ Anatomical cluster information of the gene.

^c^ Abiotic cluster information of the gene.

^d^ Key trait of characterized role of the gene.

^e^ Title of research paper of the gene.

^f^ Biotic or abiotic stress related trait.

^g^ Morphological trait.

^h^ Physiological trait.

## Data Availability

The data used to support the findings of this study are available from the corresponding author upon request.

## Supplementary Materials

Supplementary 1 Table S1. List of 3,314 plastid-localized genes. Table S2. Anatomical expression data and cluster information of 1,695 plastid-related genes. Table S3. Abiotic stress expression data and cluster information of 264 plastid-related genes.

Supplementary 2 Figure S1. KMC clustering analysis for anatomical expression data of 3,314 genes using the Affymetrix array.

Supplementary 3 Figure S2. Detailed Gene Ontology (GO) and Kyoto Encyclopedia of Genes and Genomes (KEGG) enrichment analysis of 1,695 leaf-preferred or ubiquitously expressed genes.

Supplementary 4 Figure S3. Abiotic stress clusters for 1,695 genes using Affymetrix array.
