# Supplementary figures and images for: A Systematic View Exploring the Role of Chloroplasts in Plant Abiotic Stress Responses
Source: Biomed Res Int. 2019 Jul 18;2019:6534745. doi: 10.1155/2019/6534745 (PMC6668530; doi:10.1155/2019/6534745)

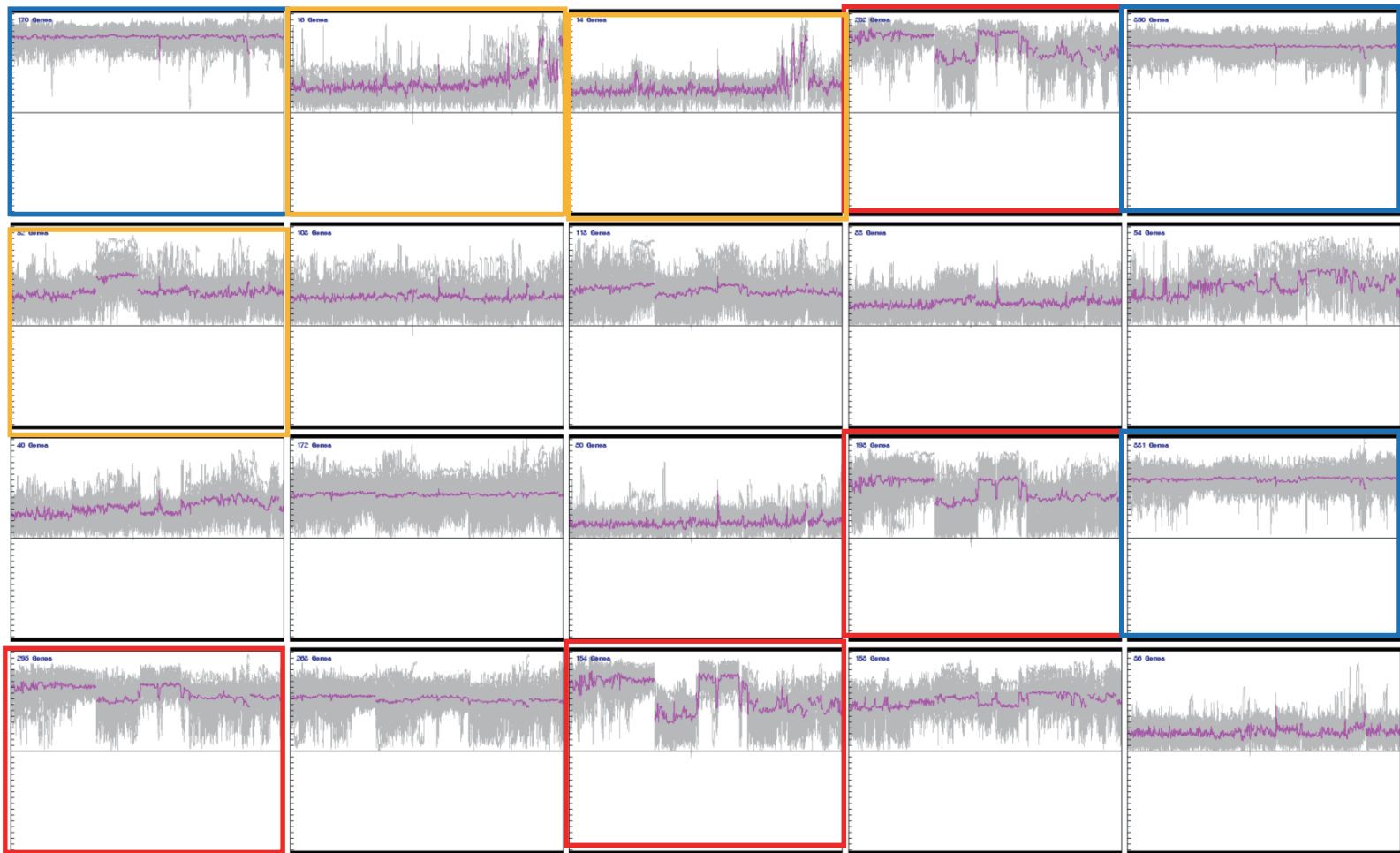

Leaf specific  
(4, 14, 16, 18)

Tissue specific  
(Seed / Anther /  
Root)

Ubiquitous  
(1,5,15)

Supplement: Supplementary 2 — Figure S1. KMC clustering analysis for anatomical expression data of 3,314 genes using the Affymetrix array. [file 6534745.f2.pdf]

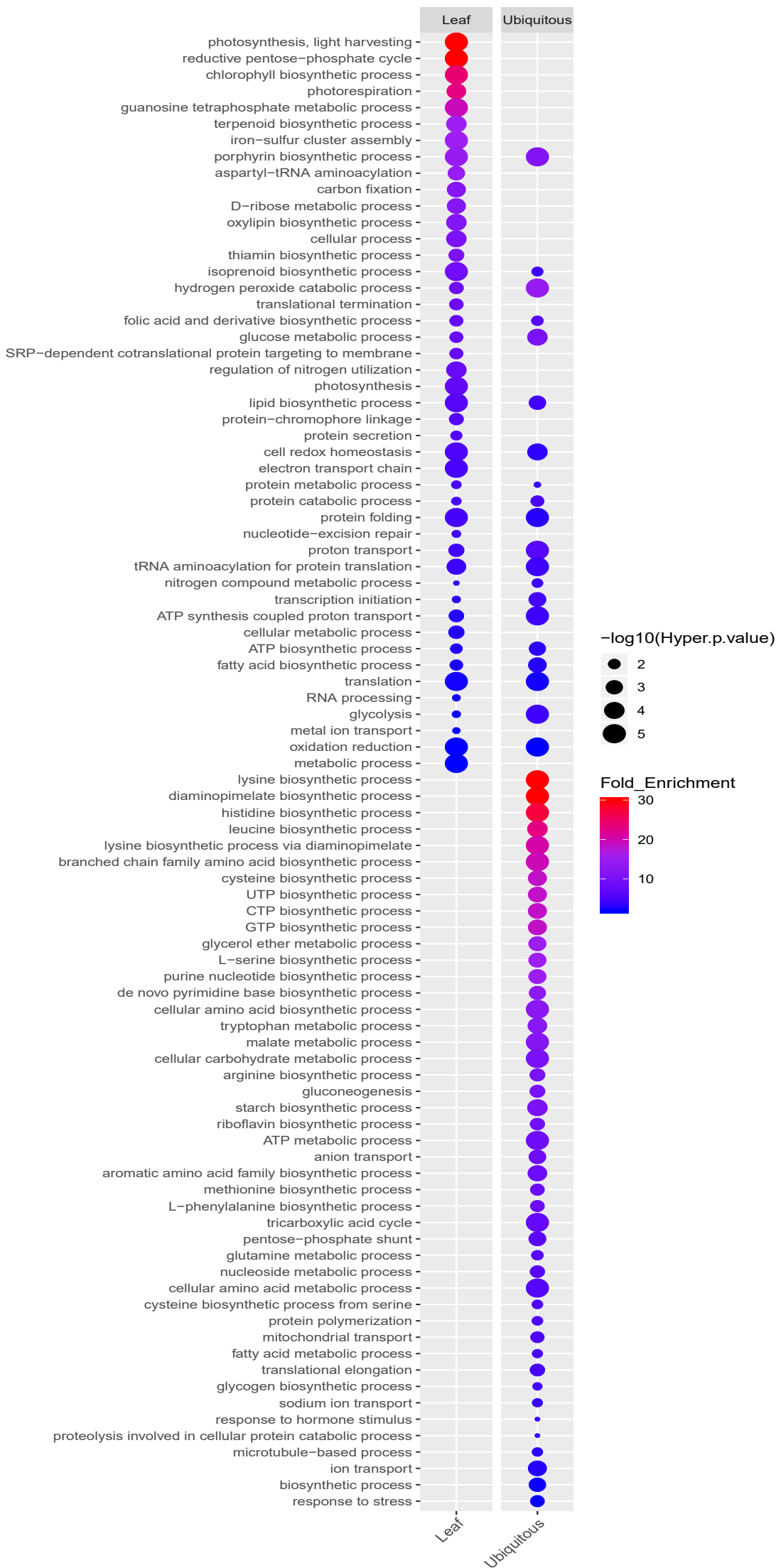

Supplement: Supplementary 3 — Figure S2. Detailed Gene Ontology (GO) and Kyoto Encyclopedia of Genes and Genomes (KEGG) enrichment analysis of 1,695 leaf-preferred or ubiquitously expressed genes. [file 6534745.f3.pdf]

Cold  
(1)

Heat  
(2, 3)

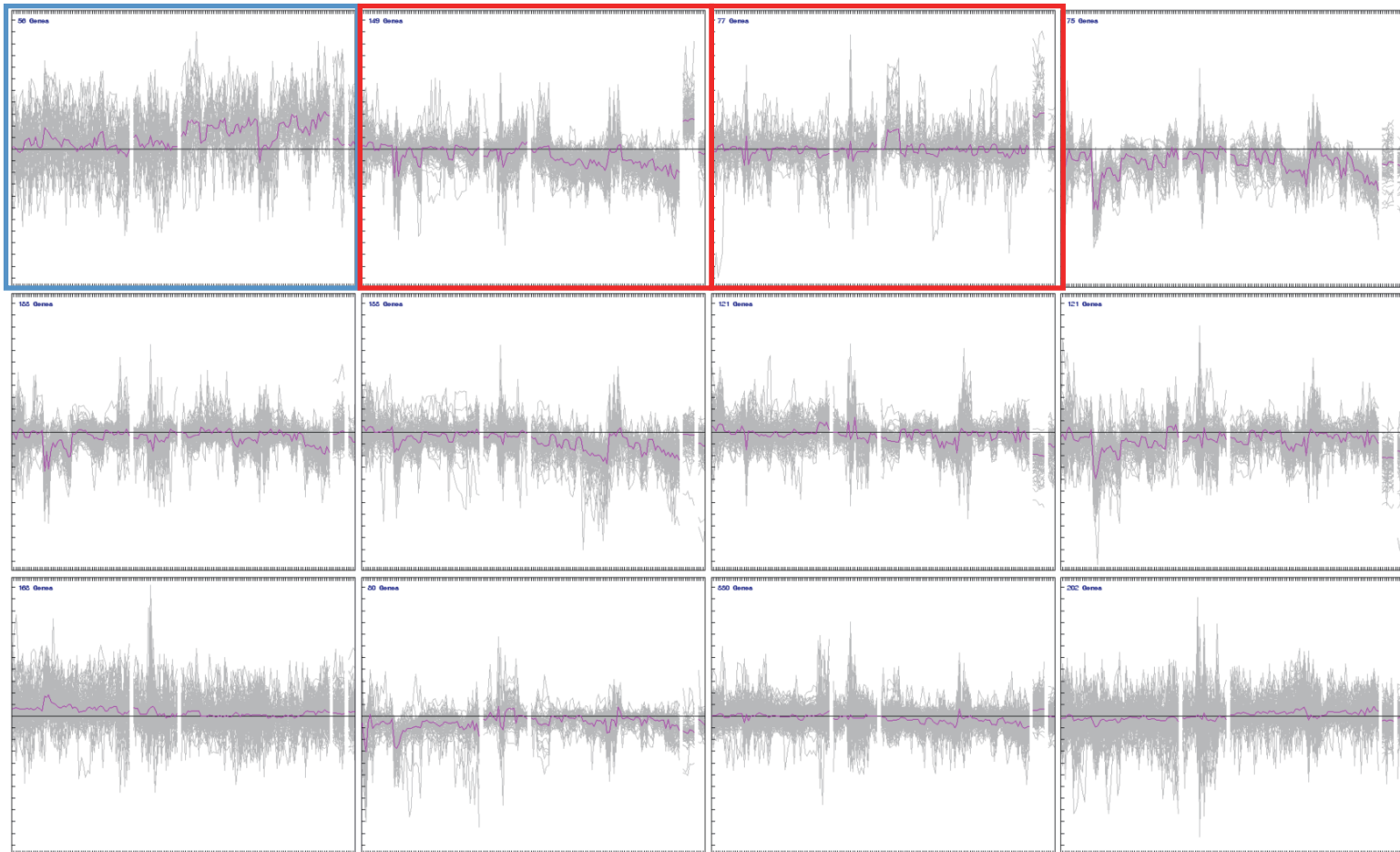

Supplement: Supplementary 4 — Figure S3. Abiotic stress clusters for 1,695 genes using Affymetrix array. [file 6534745.f4.pdf]
